# Genomic and functional portrait of multidrug-resistant, hydrogen sulfide (H_2_S)-producing variants of *Escherichia coli*

**DOI:** 10.3389/fmicb.2023.1206757

**Published:** 2023-07-27

**Authors:** Razib Mazumder, Arif Hussain, Mohammad Mustafizur Rahman, Jody E. Phelan, Susana Campino, Ahmed Abdullah, Taane G. Clark, Dinesh Mondal

**Affiliations:** ^1^Laboratory Sciences and Services Division, International Centre for Diarrhoeal Disease Research Bangladesh (icddr,b), Dhaka, Bangladesh; ^2^Department of Biotechnology and Genetic Engineering, Jahangirnagar University, Dhaka, Bangladesh; ^3^Department of Infection Biology, London School of Hygiene and Tropical Medicine, London, United Kingdom; ^4^Department of Infectious Disease Epidemiology, London School of Hygiene and Tropical Medicine, London, United Kingdom

**Keywords:** atypical H2S-producing *E. coli* variants, genomics, epidemiology, functional characterization, differential diagnosis, *E. coli* identification algorithm

## Abstract

Atypical *Escherichia coli* forms exhibit unusual characteristics compared to typical strains. The H_2_S-producing variants of some atypical *E. coli* strains cause a wide range of illnesses in humans and animals. However, there are sparse reports on such strains worldwide. We performed whole-genome sequencing (WGS) and detailed characterization of four H_2_S-producing *E. coli* variants from poultry and human clinical sources in Dhaka, Bangladesh. All four isolates were confirmed as *E. coli* using biochemical tests and genomic analysis, and were multidrug-resistant (MDR). WGS analysis including an additional Chinese strain, revealed diverse STs among the five H_2_S-producing *E. coli* genomes, with clonal complex ST10 being detected in 2 out of 5 genomes. The predominant phylogroup detected was group A (*n* = 4/5). The *bla*_TEM1B_ (*n* = 5/5) was the most predominant extended-spectrum beta-lactamase (ESBL) gene, followed by different alleles of *bla*_CTX-M_ (*bla*_CTX-M_ -55,-65,-123; *n* = 3/5). Multiple plasmid replicons were detected, with IncX being the most common. One *E. coli* strain was classified as enteropathogenic *E. coli*. The genomes of all five isolates harbored five primary and four secondary function genes related to H_2_S production. These findings suggest the potential of these isolates to cause disease and spread antibiotic resistance. Therefore, such atypical *E. coli* forms should be included in differential diagnosis to understand the pathogenicity, antimicrobial resistance and evolution of H_2_S-producing *E. coli.*

## Introduction

*Escherichia coli* is one of the most genetically diverse and versatile organisms, varying from commensal/avirulent to highly specialized pathogenic strains. *E. coli* can thrive in several niches, including hosts and in the environment (Kaper et al., 2004; Braz et al., 2020). The variant strains of *E. coli* may act as facultative or obligate pathogens (Köhler and Dobrindt, 2011). The facultative strains of pathogenic *E. coli* survive in the intestinal tract and often cause opportunistic infections when reaching suitable extraintestinal sites (Nataro and Kaper, 1998; Kaper et al., 2004). In contrast, enteric obligate pathogens can cause infections in different conditions that range from moderate to severe diarrhea, and can sometimes cause lethal gastrointestinal infections (Nataro and Kaper, 1998).

Pathogenic variants of *E. coli* are responsible for infections in a variety of animals, more commonly in humans and poultry (Bélanger et al., 2011; Hussain et al., 2017). Pathogenic *E. coli* has been reported in livestock, including poultry, cattle, and swine (Bélanger et al., 2011). Animal reservoirs of pathogenic *E. coli* are responsible for diseases in animals, but can spread the infections to humans, including antimicrobial resistant (AMR) strains (Bélanger et al., 2011). Traditionally, biochemical tests have been used for differentiating and identifying members of Enterobacteriaceae, including hydrogen sulfide (H_2_S) gas (Zabransky et al., 1969). H_2_S can be synthesized by bacteria such as *Campylobacter*, *Salmonella*, *Citrobacter*, and *Erwardsiella* and *Proteus* species on TSI or KIA media (Blachier et al., 2019). This distinct characteristic feature of H_2_S production by certain bacteria within Enterobcateriacea is used as a bacterial identification test in diagnostic microbiology. *E. coli* generally does not produce H_2_S, which differentiates it from the other members of Enterobacteriaceae (Percival et al., 2004). However, a few studies worldwide have reported the presence of atypical H_2_S-producing *E. coli* forms in humans and animals (Darland and Davis, 1974; Maker and Washington, 1974; Magalhães and Vance, 1978). They have been isolated from poultry, swine and clinical human urine specimens.

The enzyme 3-mercaptopyruvate sulfurtransferase (3MST) is reported to be mainly responsible for the synthesis of endogenous H_2_S in Enterbacteriaceae (Mironov et al., 2017). Some studies have also demonstrated the transmissibility of H_2_S-producing traits between strains via plasmids (Jones and Silver, 1978; Magalhães and Vance, 1978). Although the physiological function of endogenously produced hydrogen sulphide is not clearly defined, recent studies have pointed out its role in protecting bacteria against antibiotics and host defence systems (Mironov et al., 2017; Rahman et al., 2020). A plausible explanation for this is that the antibiotics induce oxidative stress in bacteria by increasing the levels of reactive oxygen species; in response to this, the bacteria produces H_2_S which in turn stimulates enzymes such as superoxide dismutase (SOD) and catalase that alleviates the effect of reactive oxygen species, and thereby reduces the efficacy of antibiotics contributing to AMR (Eswarappaápradeep, 2017). Also, studies have demonstrated the role of bacterial H_2_S production in defence against host immunity by making them resistant to leukocytes- mediated killing *via* unknown mechanisms (Toliver-Kinsky et al., 2019; Rahman et al., 2020).

The accurate identification of H₂S-producing variants of *E. coli* in diagnostic laboratories is an important step for initiating effective infection management. There is a need to raise awareness of this unusual type of *E. coli* form that occurs frequently but differs in its inability to produce H_2_S compared to the typical *E. coli* forms. Therefore, this study aimed to perform bacteriological, biochemical and genomic characterization of H_2_S-producing variants of *E. coli* from healthy poultry and human clinical sources in Dhaka, Bangladesh. We present the first report on the genomic characterization of H_2_S-producing variants of *E. coli* from Bangladesh and that from South Asia.

## Materials and methods

### Ethics statement

The study protocols were approved by the Research Review committee and Ethics Review Committee of icddr,b, Dhaka, Bangladesh (PR-23045).

### Bacterial strains

A surveillance study was conducted between 2019 and 2021 to investigate the genomic-based epidemiology of AMR Enterobacteriaceae in healthy poultry and human clinical samples in Dhaka, Bangladesh (Mazumder et al., 2020a, 2021, 2022). During that study, we detected four lactose fermenting *E. coli* colonies but with an atypical biochemical feature of H_2_S production. These were confirmed to be *E. coli* by the methods described hereafter. Three (BD7, BD8, BD9) of these H_2_S-producing *E. coli* originated from raw poultry meat and one isolate (BD_CL10) was cultured from a urine sample of a suspected urinary tract infection patient in Dhaka, Bangladesh. Thus, from a collection of 96 poultry *E. coli* isolates and 204 human clinical *E. coli* study isolates, we could obtain three and one H_2_S-producing *E. coli* isolates, respectively. These four H_2_S-producing *E. coli* isolates then formed the basis of this study, and underwent various tests and whole genome sequencing (WGS). One H_2_S positive *E. coli* genome from China (Biswas et al., 2020) was used for the in-silico analysis together with the four studied H_2_S positive *E. coli* genomes sequenced in this study.

### Biochemical characterization and antimicrobial susceptibility

The complete bacteriological and biochemical characteristics of H_2_S-producing variants of *E. coli* strains are summarized (Table 1). The biochemical identification included the following tests; kligler iron agar (KIA) test, motility, indole and urease (MIU) test, citrate and acetate utilization test, catalase test, oxidase test, vogas-proskauer test, gelatin liquefaction and ONPG tests. In addition, colonies were plated on Muller-Hinton agar containing 0.68% of sodium thiosulfate plus 0.08% of ferric ammonium sulfate as previously described (Park et al., 2015). The isolates that mimic *E. coli* in all aspects except H_2_S-production in Kligler iron agar (KIA) and Muller-Hinton agar (with sodium thiosulfate and ferric ammonium sulfate) were carried forward in this study. These preliminary identified 4 H_2_S-positive *E. coli* isolates were subjected to additional tests, including fermentation of sugars and decarboxylation reaction of amino acids (Mazumder et al., 2022). Further, the possibility of *Salmonella* spp. was ruled out by slide agglutination test using O, O1 polyvalent and VI *Salmonella* antisera (Denka Seiken Co. Ltd. Tokyo, Japan). The API 20E kit (bioMérieux) was used to generate the analytical profile index (Table 1). Haemolysis was evaluated using 5% sheep blood agar plates. Disk diffusion method was employed to determine the antimicrobial susceptibility. The Clinical and Laboratory Standards Institute (CLSI) guidelines (Weinstein, 2019) were followed. Twenty commercially available antibiotic disks (Oxoid, US) covering 11 antimicrobial classes were tested (see Table 2). The intermediate susceptibility was described as non-susceptible. Isolates were termed multi-drug resistant (MDR) if refractory to at least one antibiotic from three or more antimicrobial classes (Magiorakos et al., 2012).

**Table 1:** Biochemical and growth characteristics of H_2_S-producing *Escherichia coli* from Dhaka, Bangladesh.

Biochemical tests performed	Test results			
	Isolate BD7	Isolate BD8	Isolate BD9	BD_CL10
Gram stain	Gram negative bacilli	Gram negative bacilli	Gram negative bacilli	Gram negative bacilli
Catalase test	+	+	+	+
Oxidase test	−	−	−	−
**TSI agar**				
a. Acid production in slant	+	+	+	+
b. Acid production in butt	+	+	+	+
c. Hydrogen sulfide production (H_2_S)	+	+	+	+
d. Gas production	+	+	+	+
**Motility indole ureas test (MIU)**				
a. Motility	+	+	+	+
b. Indole Production	+	+	+	+
c. Urea hydrolysis	−	−	−	−
Simmons citrate reaction test	−	−	−	−
Acetate	+	+	+	+
Mueller Hinton agar + sodium thiosulfate with ferric ammonium sulfate	Produce H_2_S	Produce H_2_S	Produce H_2_S	Produce H_2_S
**Sugar fermentation**				
a. Glucose	+	+	+	+
b. Lactose	+	+	+	+
c. Sucrose	+	+	−	+
d. Maltose	+	+	+	+
e. Mannose	+	+	+	+
f. Arabinose	+	+	+	+
g. Sorbitol	+	+	+	+
h. Mannitol	+	+	+	+
i. Inositol	−	−	−	−
Nitrate Reduction	+	+	+	+
Gelatine liquefaction	−	−	−	−
ONPG	+	+	+	+
Vogas-proskauer	−	−	−	−
Lysine decarboxylase	+	+	+	+
Ornithine decarboxylase	−	−	+	−
Arginine dihydrolase	+	+	−	+
Haemolysis on blood agar	−	−	−	−
**Growth characteristics**				
a. MacConkey agar	PC^a^	PC^a^	PC^a^	PC^a^
b. SS agar agar	PC^a^	PC^a^	PC^a^	PC^a^
c. CHROMagar™ Orientation	DPC^b^	DPC^b^	DPC^b^	DPC^b^
d. Blood agar	WC^c^	WC^c^	WC^c^	WC^c^
e. Gelatin agar	WC^c^	WC^c^	WC^c^	WC^c^
Growth Temperature	26–42°C	26–42°C	26–42°C	26–42°C
API Number (Detect *E. coli* with 99% probability)	5,544,512	5,544,512	5,544,552	5,544,512

a PC, pink color colony.

^b^DPC = Dark pink color colony.

^c^WC = White colony.

**Table 2:** Antimicrobial susceptibility profiles of H_2_S-producing *Escherichia coli* isolates from Dhaka, Bangladesh.

Classes	Antibiotics	BD7	BD8	BD9	BDCl-10	China_H2S
Aminoglycosides	Amikacin (AK)-30 μg	S	S	S	S	DA
	Gentamicin (CN)-10 μg	R	S	R	S	R
*β*-Lactams (Penicillin)	Ampicillin (Amp)-10 μg	R	R	R	R	R
*β* Lactams (Cephalosporins)	Cefepime (FEP)-30 μg	S	R	R	R	DA
	Cefixime (CFM)-5 μg	S	R	R	R	DA
	Cefotaxime (CTX)-30 μg	S	R	R	R	S
	Ceftazidime (CAZ)-30 μg	S	R	R	R	DA
	Ceftriaxone (CRO)-30 μg	S	R	R	R	DA
	Cefuroxime (CXM)-30 μg	S	R	R	R	R
Phenicols	Chloramphenicol (C)-30 μg	R	S	R	S	R
Fluoroquinolones	Ciprofloxacin (CIP)-5 μg	R	R	R	R	R
	Nalidixic Acid NA-30 μg	R	R	R	R	R
Polymyxins	Colistin (CT)-10 μg	13.7*	13.8*	13.8*	13.6*	R
Trimethoprim/Sulfonamides	Trimethoprim- sulfamethoxazole (SXT)-1.25/ 23.75 μg	R	R	R	R	R
Tetracyclines	Doxycycline (DO)-30 μg	R	R	R	R	R
Phosphonic antibiotic	Fosfomycin (FOS)-50 μg	R	S	R	I	S
Carbapenems	Imipenem (IPM)-10 μg	S	S	S	S	S
	Meropenem (MEM)-10 μg	S	S	S	S	S
Nitrofuran derivatives	Nitrofurantoin (F)300 μg	R	S	I	I	DA
Glycylcycline	Tigecycline (TGC)-15 μg	R	R	R	R	DA

R, resistance; S, susceptible, I, intermediate, DA, data absent, *zone of inhibition in mm.

### Whole-genome sequencing

Total bacterial DNA was extracted using the Maxwell RSC Instrument and Culture Cell DNA extraction Kit (Promega) for gram-negative bacteria with an additional RNaseA treatment (Baddam et al., 2020; Mazumder et al., 2020b,c). The DNA QC was assessed by Nanodrop spectrophotometer (Thermo Fisher Scientific, US), Quantus Fluorometer (Promega, US) and by 1% agarose gel electrophoresis. The paired-end bacterial WGS libraries were constructed from 220 to 250 ng of genomic DNA using the Illumina DNA Prep kit as per the manufacturer’s instructions (Mazumder et al., 2021). The pooled libraries thus obtained were sequenced at the icddr,b Genome Centre on Illumina NextSeq 500 system to obtain 100- to 150-fold coverage for each genome using a NextSeq v2.5 Mid Output reagent kit (2 × 150 bp) (Mazumder et al., 2022; Monir et al., 2023).

### Sequence assembly and annotation

WGS data quality was examined using FastQC (Andrews, 2010). Trimmomatic software (v0.36) (Faircloth, 2013) was used to extract adapters and poor-quality bases (<Q30) from the unprocessed sequencing reads using the following parameters described elsewhere (Mazumder et al., 2020c, 2021, 2022). Deconseq software (v4.3) was used to eliminate contaminated sequences (Schmieder and Edwards, 2011). The processed reads were used to create *de novo* assemblies of each genome using SPAdes software (v3.11.1) (Bankevich et al., 2012). QUAST (v5.0) (Gurevich et al., 2013) was used to evaluate the assembly metrics of scaffold fasta files. The genomes were annotated using Prokka (v1.12) (Seemann, 2014) using *E. coli* MG1655 as the reference genome (GenBank accession number NC 000913.3). The genomic features of H_2_S-producing *E. coli* strains are summarized (Table 3).

**Table 3:** Genomic features, the status of CRISPR-CAS system and prophage sequences in H_2_S-producing *Escherichia coli* isolates.

Strain Name		BD7	BD8	BD9	BD-Cl10	H_2_S *E.coli*_China
Pathogenicity Score (No. of Pathogenic Families)		0.94 (666)	0.93 (635)	0.933 (585)	0.941 (548)	0.934 (567)
Human Pathogenicity		Yes	Yes	Yes	Yes	Yes
Genomic features of H_2_S producing *E. coli* isolates	Genome Size (bp)	5,050,301	5,198,676	4,990,709	4,525,004	4,501,832
	Genome coverage	102X	127X	125X	133X	206X
	Contig no. (>500 bp)	149	248	135	125	122
	GC %	50.37%	50.43%	50.63%	50.87%	50.71%
	No. of Coding Sequences	4,809	5,069	4,757	4,224	4,216
	Accession No.	JAGINC000000000	JAGIND000000000	JAGINE000000000	JAODTH000000000	Not found
	SRA	SRX11616412	SRX11616413	SRX11616414	SRX17654297	SRX6956426
	Bio-project	**PRJNA714244**			**PRJNA882002**	**PRJNA576077**
Characteristic features of CRISPR-Cas system	Subtype	I-E, I-A	I-A, I-E	I-A, I-E	I-A, I-E	I-A
	Cas Proteins	Cas3, DEDDh, Csa3, Cas8e, Cse2gr11, Cas7, Cas5, Cas6e, Cas1, Cas2	Cas3, DEDDh, Csa3, Cas8e, Cse2gr11, Cas7, Cas5, Cas6e, Cas1, Cas2	Cas8e, DEDDh, Cas3, Cas2, Cas1, Cas6e, Cas5, Cas7, Cse2gr11, Cas3	Cas3, DEDDh, Csa3, Cas8e, Cse2gr11, Cas7, Cas5, Cas6e, Cas1, Cas2	Csa3, DEDDh, Csa3
	No. of loci	1	1	1	1	1
	No. of repeats	12	14	7	19	5
	Average length of repeats	29	29	29	29	29
	No. of spacers	11	13	6	18	4
	Average length of spacers	32	32	32	32	32
	Questionable CRISPR*	+	+	+	+	+
Completeness of prophage sequences#	Intact	2	3	3	4	0
	Incomplete	4	8	7	1	5
	Questionable	2	2	0	0	0
	Total prophage regions	**8**	**13**	**10**	**5**	**5**
	Intact prophage Region Length	26.9Kb; 37.6Kb	26Kb, 34.8Kb, 12.3Kb	49.7Kb, 46.8Kb, 100.2Kb	38.6Kb, 32.3Kb, 39.2Kb, 35.5Kb	ND^a^
Intact Phage Name based on highest number of hits		Enterobacteria phage SfI-13 Klebsiella phage 4 LV-2017	Yersinia phage L413C Shigella phage SfII Enterobacteria phage HK544	Enterobacteria phage P88 Salmonella phage118970_sal3 Salmonella phage SSU5	Enterobacteria phage Lambda Klebsiella phage 4 LV-2017 Escherichia phage 500,465–1 Shigella phage SfII	ND^a^

ND^a^, not detected.

### *In silico* sequence analysis

The reads of H_2_S-producing *E. coli* were uploaded to the KmerFinder v3.2 (Hasman et al., 2014; Larsen et al., 2014) for species confirmation. The phylogenetic groups were ascertained using Clermon Typing tool (Beghain et al., 2018). The sequence types (STs), clonal complex and pathovars were predicted employing the Achtman7 seven-locus scheme at EnteroBase v1.1.3^1^ web tool. The O and H serotypes were determined employing SerotypeFinder v2.0 (Joensen et al., 2015). FimH and FumC types were determined by CH typer 1.0 (Roer et al., 2018). AMR determinants, virulence factors, and plasmid types were screened using the ABRicate tool v1.0.1 (Seemann, 2018), ResFinder (Zankari et al., 2012), Virulence Factor Database (VFDB) (Chen et al., 2005), and PlasmidFinder (Carattoli et al., 2014) databases, respectively. We used a cut-off of 80% query coverage and 98% identity for screening genes in the genomes analysed. Mobile Element Finder (v1.0.3) was utilized to identify mobile genetic elements linked with acquired antimicrobial resistance genes. Mutations encoding fluoroquinolone resistance were detected by PointFinder (Zankari et al., 2017). IntegronFinder (v2.0) was used to identify integrons (Néron et al., 2022). The chromosomal or plasmid origin of ESBLs genes were analysed by *BLA*STn analysis of contigs against NCBI database. Prophage sequences in *E. coli* genomes were determined using the Phage Search Tool Enhanced Release (PHASTER). Prophage regions were classified as intact, questionable, and incomplete based on prophage sequence scores of ≥90, 70–90, and ≤ 70, respectively. (Arndt et al., 2016). CRISPR-Cas system of H_2_S-producing *E. coli* strains were characterized using CRISPRone tool^2^ (Zhang et al., 2012). Cysteine-degradation genes in *E. coli* were identified based on genes described previously (Braccia et al., 2021). A threshold of 100% coverage and 98% identity were used. The pathogenic pontential of strain was predicted using the web-server PathogenFinder (Cosentino et al., 2013). Default parameters were used for the *in-silico* analysis unless otherwise stated.

### Single nucleotide polymorphism-based core genome phylogeny

We used Snippy (v4.4.0) software (Seemann, 2015) with default parameters to obtain the reference-guided multi-fasta consensus alignment of 5 H_2_S-producing *E. coli* genomes using *E. coli* MG1655 as the reference. Gubbins software (v3.2 5) (Croucher et al., 2015) was used to filter true point mutations from those arising from recombination. The phylogenetic tree was determined using RaxML (v8.2.12), utilizing the Generalized Time Reversible substitution model and a GAMMA distribution to account for rate heterogeneity (Stamatakis, 2014). Finally, the phylogenetic tree was displayed using IToL (Letunic and Bork, 2016).

### Accession numbers

The four genomes that were sequenced for this study can be identified by their GenBank accession numbers: JAGINC000000000 (BD7), JAGIND000000000 (BD8), JAGINE000000000 (BD9) and JAODTH000000000 (BD_CL10) (Table 3).

## Results

### Bacterial characteristics

**Figure 1:**
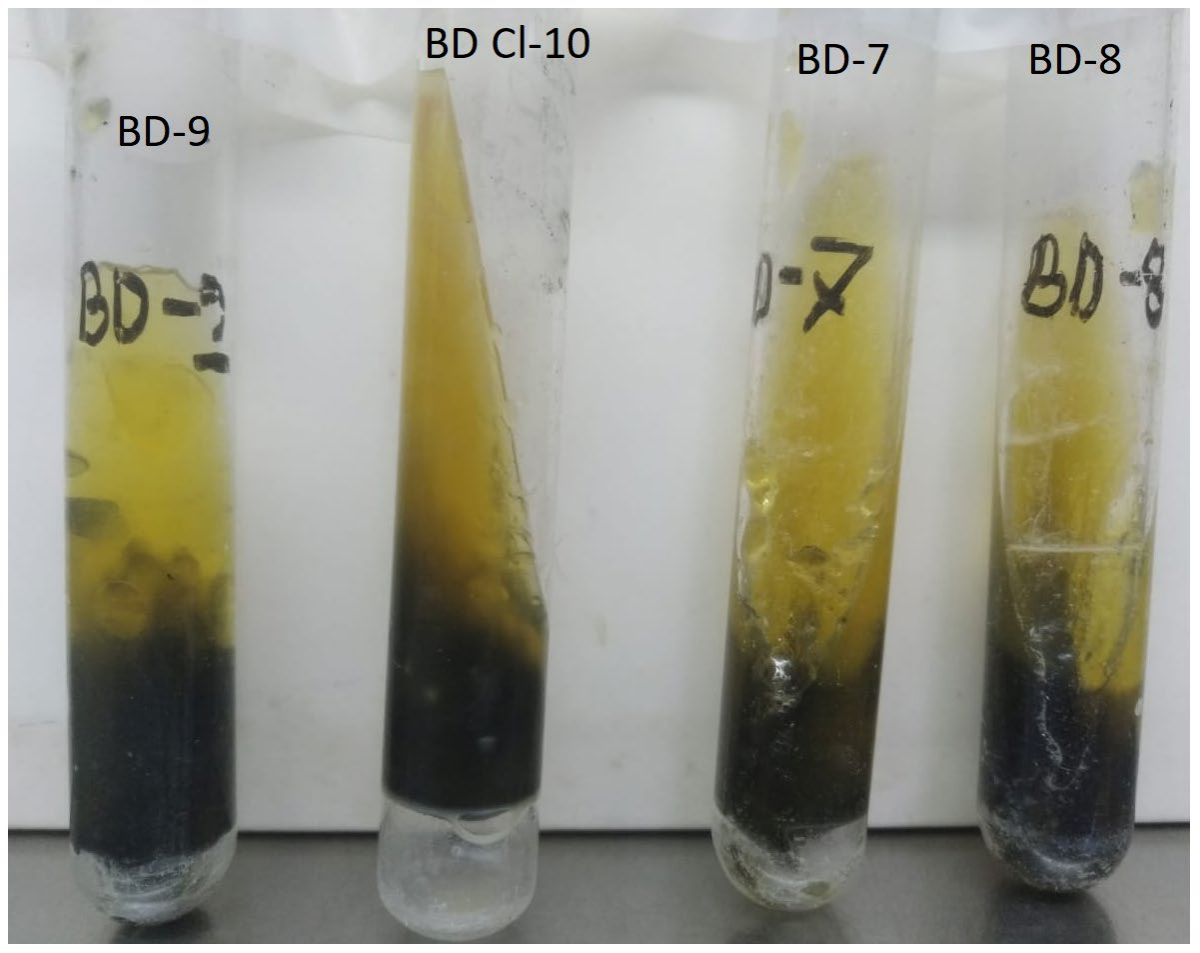
Hydrogen Sulfide (H_2_S)-producing variants of *Escherichia coli* strains: BD7, BD8, BD9, and BD-CL10 showing black precipitate of H_2_S on Kligler Iron Agar (KIA) tubes.

Four H_2_S-positive *E. coli* variants were identified that formed a black precipitate after overnight incubation in an aerobic environment in the Kligler iron agar (KIA) and Mueller Hinton agar medium enriched with both sodium thiosulfate and ferric ammonium sulfate (Figure 1). Attempts to agglutinate the strains with polyvalent *Salmonella* antisera yielded negative results. When streaked on CHROMagar Orientation media, *E. coli* produced small, pink-red colonies that were characteristic of the species. Routine biochemical tests identified the strains as *E. coli*, except for their ability to reduce thiosulfate to H_2_S (Table 1). All four isolates were gram-negative rods, motile, oxidase-negative, catalase-positive and indole positive. All isolates tested showed a lack of urease and Voges-Proskauer reaction, and they did not grow on the Simmons Citrate agar medium. Nonetheless, all isolates exhibited a positive result for O-nitrophenyl-beta-D-galactopyranoside (ONPG) and carried out fermentation of glucose and lactose sugars, leading to gas production (Figure 1). The API results revealed two distinct profiles, 5,544,512 (*n* = 3), and 5,544,552 (*n* = 1) and confirmed isolates as *E. coli* with 99% certainty (Table 1). The optimum growth temperate ranged between 26° to 42°C and they produced gamma-haemolysis on sheep blood agar.

### Molecular and phylogenomic analysis of H_2_S-positive *E. coli* genomes

**Figure 2:**
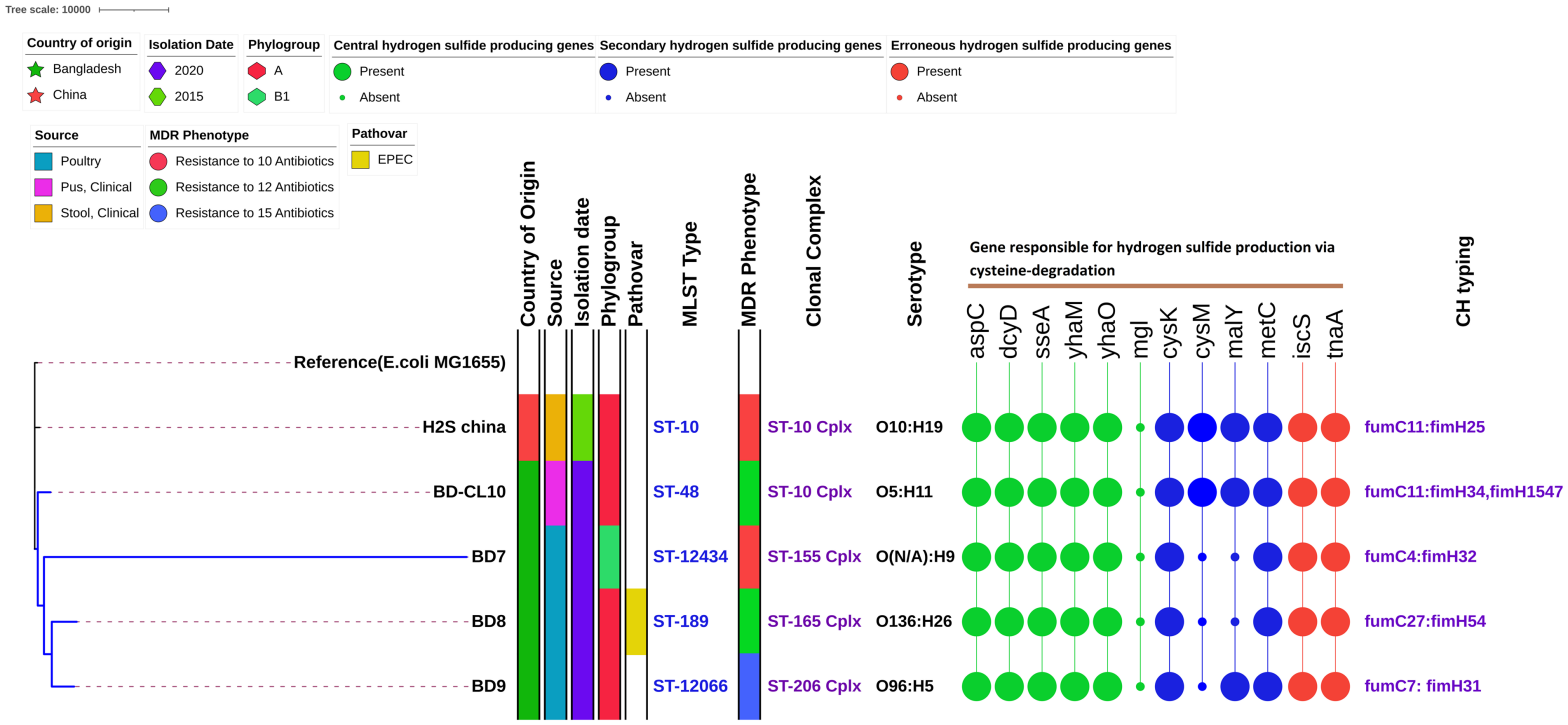
Phylogenetic relationships among sequenced Hydrogen Sulfide (H_2_S)-Producing *Escherichia coli* genomes. The maximum likelihood phylogenetic tree is based on the alignment of detected core genomes with the MG1655 genome strain as a reference genome. The country of origin, isolation date, sample source, phylogroups, pathovar, MDR status, STs, clonal complex, serogroups, genes responsible for hydrogen sulfide production via cysteine-degradation and CH types are shown next to the tree.

This analysis included the four in-house strains and a genome of H_2_S-producing *E. coli* reported from China (China_H_2_S). WGS-based species identification confirmed all the isolates as *E. coli*. Across the five H_2_S-producing *E. coli* strains, the average genome size was 4,853,304 bp (range 4,501,832 to 5,198,676) with an average GC content of 50.6% (range: 50.4 to 50.9%). The genome assemblies had an average coverage of 138-fold, with a range of 102 to 206-fold (Table 3). They had five distinct STs, which comprised ST10, ST48, ST12434, ST189, and ST12066. We detected four clonal complexes that included CC10 (two strains from human sources) followed by CC155, CC165 and CC206, representing one strain each (Figure 2). We identified four isolates (80%) belonging to commensal phylogroup A and one isolate (20%) to B1 phylogroup. All H_2_S-producing *E. coli* isolates exhibited distinct serotypes and CH types. A phylogenetic tree was constructed for five H2S-producing *E. coli* genomes using the MG1655 genome as a reference, by aligning the core genome single nucleotide polymorphisms (SNPs). The studied H_2_S-producing *E. coli* strains were found to be relatively diverse. However, the three poultry H_2_S-positive strains from Bangladesh clustered together, with human strains adjacent to this cluster. The molecular characteristics did not correlate with the source of origin or the phylogenetic clustering of the H_2_S-producing *E. coli* isolates (Figure 2).

### H_2_S-producing genes

All five H_2_S-producing *E. coli* genomes harbored five primary hydrogen sulfide-producing genes; cysteine aminotransferase (*aspC*), cysteine desulfhydrase (*dcyD*), 3-mercapto pyruvate sulfurtransferase (*sseA*), yhaOM operon (*yhaM, yhaO*). Whereas the methionine gamma-lyase (*mgl*) gene was completely absent. Two of the four secondary function genes, cysteine synthase A (*cysK*) and cystathionine beta-lyase (*metC*) were found in all four strains. However, the other genes such as cysteine synthase B (*cysM*), and cystathionine beta-lyase-like repressor of maltose regulon (*malY*) are sparingly present in poultry isolates. The erroneous H_2_S-producing genes, including cysteine desulfurase (*iscS*) and tryptophanase (*tnaA*) were observed in all isolates. As expected, all three class of cysteine-degradation genes were found on chromosomes (Figure 2; Table 4).

**Table 4:** Cysteine-degradation genes and their location in the H_2_S-producing *Escherichia coli* genomes.

Cysteine-degradation based H2S producing Gene		Genome locus				
		BD7	BD8	BD9	BD-Cl 10	China_H2S
*aspC*	Cysteine aminotransferase	Chromosome	Chromosome	Chromosome	Chromosome	chromosome
*dcyD*	Cysteine desulfhydrase	Chromosome	Chromosome	Chromosome	Chromosome	chromosome
*sseA*	3-mercaptopyruvate sulfurtransferase	Chromosome	Chromosome	Chromosome	Chromosome	chromosome
*yhaM*	yhaOM operon	Chromosome	Chromosome	Chromosome	Chromosome	chromosome
*yhaO*		Chromosome	Chromosome	Chromosome	Chromosome	chromosome
*mgl*	Methionine gamma-lyase	Absent	Absent	Absent	Absent	Absent
*cysK*	Cysteine synthase A	Chromosome	Chromosome	Chromosome	Chromosome	chromosome
*cysM*	Cysteine synthase B	Absent	Absent	Absent	Chromosome	chromosome
*malY*	Cystathionine beta-lyase like; repressor of maltose regulon	Absent	Absent	Chromosome	Chromosome	chromosome
*metC*	Cystathionine beta-lyase	Chromosome	Chromosome	Chromosome	Chromosome	chromosome
*iscS*	Cysteine desulfurase	Chromosome	Chromosome	Chromosome	Chromosome	chromosome
*tnaA*	Tryptophanase	Chromosome	Chromosome	Chromosome	Chromosome	chromosome

### Plasmid replicon types

PlasmidFinder identified 20 unique plasmid replicon groups (Table 5). All five isolates harbored multiple plasmid replicons. The plasmid replicons identified include IncFII (pHN7A8), IncFII (pSE11), IncFIA (HI1), IncFIB (K), IncFIB (pLF82-PhagePlasmid), IncFIB (pB171), IncHI2, IncHI2A, IncI (Gamma), IncN, IncQ1, IncR, IncX2, IncX1, IncY, ColE10, ColRNAI, Col (MG828), Col (pHAD28) and p0111 (Table 5). The majority of isolates (4/5; 80%) harbored the IncX, followed by (3/5; 60%) IncF (FII, FIB, FIA), IncN and Col. The H_2_S-producing *E. coli* strains that were positive for the *bla*_CTX-M_ gene were significantly linked to IncF-type replicons (specifically FIA, FIB, and FII) and CoI plasmids.

**Table 5:** Plasmid replicon, integrons, ESBL genes and genetic context of ESBL genes in the H_2_S-producing *Escherichia coli* isolates.

Strain	Plasmid replicon	Integrons	ESBLs producing gene		
			ESBLs gene	Genome locus	MGEs
BD7	IncHI2, IncHI2A, IncN, IncQ1, IncX2, p0111	Class 1 integron	*bla*_TEM-1B_	Plasmid	–
			*bla*_TEM-106_	Plasmid	–
			*bla*_TEM-126_	Plasmid	–
			*bla*_TEM-135_	Plasmid	–
			*bla*_TEM-220_	Plasmid	–
BD8	ColE10, ColRNAI, IncFII (pHN7A8), IncFII (pSE11), IncN, IncX1, IncY	Class 1 integron	*bla*_TEM-1B_	Plasmid	ISKra4
			*bla*_CTX-M-55_	Plasmid	–
BD9	Col (MG828), Col (pHAD28), IncFIB (K), IncFIB (pLF82PhagePlasmid), IncI (Gamma), IncN, IncX2	Class 1 integron	*bla*_TEM-1B_	Plasmid	–
			*bla*_CTX-M-65_	Plasmid	–
BD-Cl 10	ColRNAI, IncFIA (HI1), IncFIB (K), IncFIB (pB171)	Class 1 integron	*bla*_TEM-1B_	Plasmid	IS6100R
			*bla*_CTX-M-123_	Chromosome	ISEcp1
H2S _China	IncR, IncX1	Class 1 integron	*bla*_TEM-1B_	Plasmid	–
			mcr-1.1	Plasmid	–

“-” = not detected.

### Prophage analysis

We detected intact prophages in the Bangladeshi H_2_S-producing *E. coli* strains, but not in the Chinese isolate. The poultry strains harbored two to three intact prophage sequences, while the human clinical strains carried four intact prophage sequences. Incomplete prophage sequences ranged from four to seven in poultry strains and one in human strains, while questionable prophage sequences ranged from zero to two in poultry strains and were absent in human strains identified in Bangladesh. However, five incomplete prophage sequences were detected in H_2_S-producing *E. coli* from China. The most common phages in the H_2_S-producing *E. coli* strains were *Klebsiella* phage 4 LV-2017 (2/5) and *Shigella* phage SfII (2/5), with both phages present together in a single H_2_S-producing human clinical *E. coli* strain (Table 3).

### CRISPR-CAS system

The CRISPR-CAS system subtype I-A and I-E were found to be the most prevalent in the five H_2_S-producing *E. coli* genomes. All H_2_S-producing *E. coli* strains obtained from both poultry and human sources had only one CRISPR locus. The number, nucleotide sequence, and average length of repeats and spacers were similar in all H_2_S-producing *E. coli* strains, but they varied in the quantity of repeats and spacer units. The human clinical strain BDCl_10 was comparable to poultry strains, except that it had a higher number of repeats and spacers than the poultry strains (as shown in Table 3).

### Virulome

**Figure 3:**
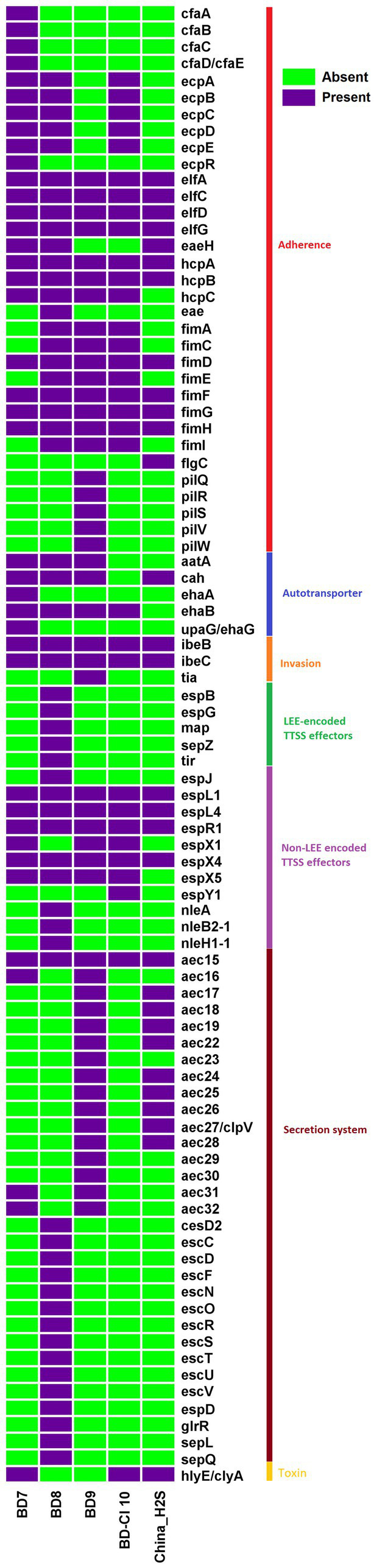
Heat map depicting the distribution of 89 virulence genes among 5 Hydrogen Sulfide (H_2_S)-producing *Escherichia coli* genomes. Dark violet blocks represent the presence, and light green blocks represent virulence gene absence.

The virulome analysis of H_2_S-producing *E. coli* isolates revealed the predominance of virulence factors (VFs) (Figure 3). The H_2_S-producing *E. coli* isolates showed a 93% mean probability of being human pathogens using the PathogenFinder web-server. The isolate BD8 harbored the highest number of VFs (57), followed by the isolates BD9 (48), BD7 (40), BD-Cl10 (32) and China_H2S (30). All isolates (5/5) harbored the type I fimbriae genes *fim* (A, C–D, E–H, l). All of the isolates showed the presence of invasin of brain endothelial cells locus B (*ibeB*) and invasin of brain endothelial cells locus C (*ibeC*) genes, which belong to the invasin virulence factor category (Figure 3). The *E. coli* laminin-binding fimbriae genes (ELF) *elfA, elfC, elfD, elfG* were also present (5/5) in all *E. coli* isolates. However, hemorrhagic *E. coli* pilus (HCP) genes associated with the production of type IV pili were highly prevalent of which *hcpA* gene was most predominant (100%, 5/5) followed by *hcpC* (80%, 4/5). Non-LEE encoded T3SS (Type III Secretion System) related genes specifically *espL1, espL4, espR1, espX1, espX4, espX5* were observed in almost all H_2_S-producing *E. coli* isolates. The autotransporter genes such as *aatA, cah, ehaB* were also prevalent (80%, 4/5) (Figure 3). The *hlyE/clyA*, a pore-forming toxin was observed in 60% (3/5) of isolates. One H_2_S-producing *E. coli* strain BD8 harbored the *Intimin* related *eae* gene and was classified as Enteropathogenic *E. coli* (EPEC). Overall, the poultry *E. coli* isolates (87) harbored higher number of VFs than human isolates (44).

### Antimicrobial resistance phenotypes and genotypes

All four (100%) H_2_S-producing *E. coli* isolates from Bangladesh were resistant to ampicillin, nalidixic acid, ciprofloxacin, sulfamethoxazole-trimethoprim, doxycycline, and tigecycline. While they were all sensitive to amikacin, imipenem, meropenem, and colistin. However, three isolates (75%) were resistant to ceftazidime, cefotaxime, cefepime, cefuroxime, and ceftriaxone. Whereas 50% of isolates were resistant to chloramphenicol, fosfomycin, and gentamicin. All four H_2_S-producing *E. coli* isolates were classified as MDR.

**Figure 4:**
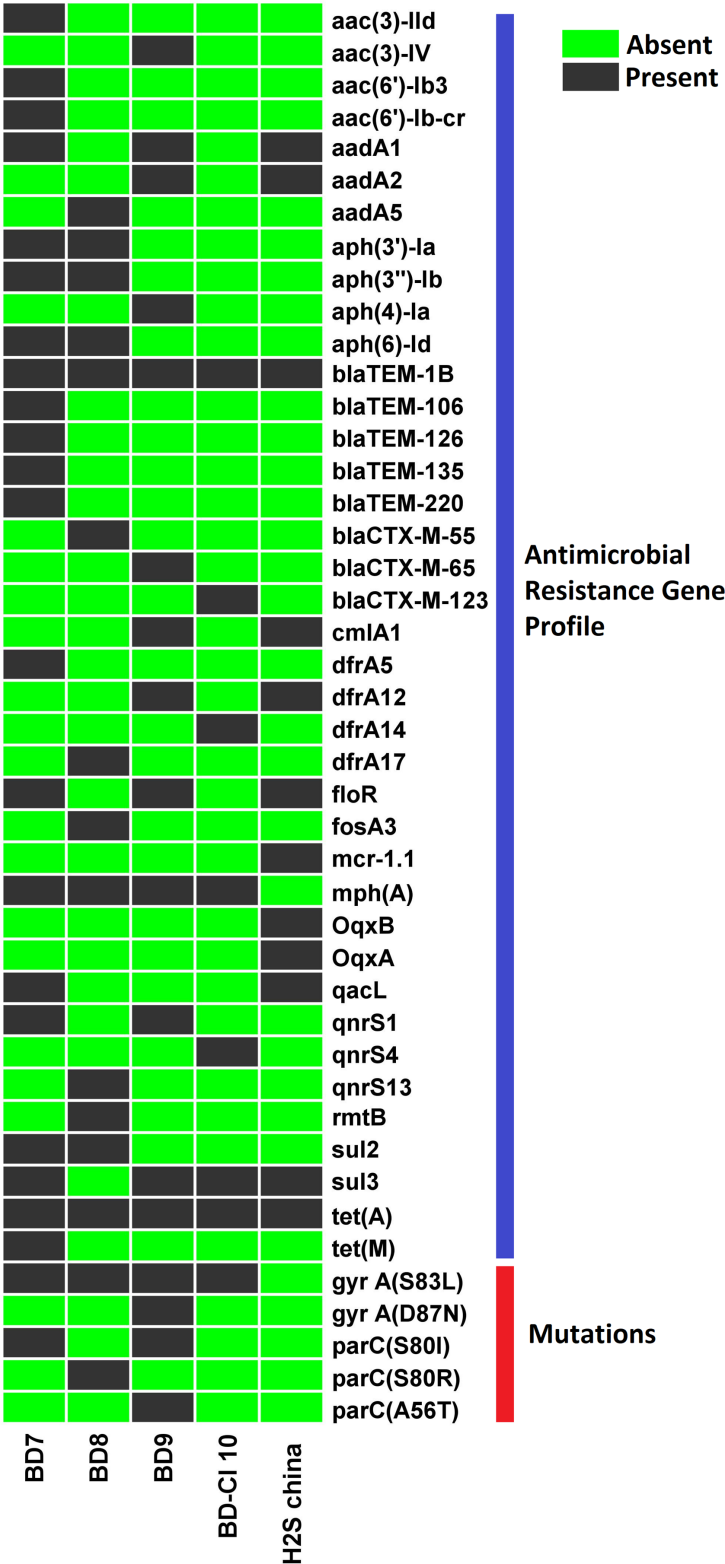
Heat map demonstrating the antimicrobial resistance (AMR) genes profile of 5 Hydrogen Sulfide (H_2_S)-producing *Escherichia coli* genomes. Dark black blocks represent the presence of AMR genes, and light green blocks represent the absence of particular genes.

We identified 43 distinct AMR gene alleles belonging to various classes (Figure 4). A minimum of seven AMR genes per genome were detected with some variation across strains (poultry *E. coli* 13–25; human *E. coli* 7–12). All (5/5) the H_2_S-producing *E. coli* genomes harbored beta-lactamase genes. All isolates were positive for the *bla*_TEM1B_ gene (100%). The *bla*_CTX-M_ gene alleles (*bla*_CTX-M-55_, *bla*_CTX-M-65_, and *bla*_CTX-M-123_) were detected in 3 out of 5 H_2_S-producing *E. coli* genomes (Figure 2). The *bla*_TEM1B_ and *bla*_CTX-M_ variants coexisted in three isolates (60%, 3/5). Among the 14 aminoglycoside resistance genes identified, *aadA1* was predominant (60%, 3/5) followed by *aadA2*, *aph* (*3′*)*-Ia*, *aph* (*3″*)*-Ib*, and *aph* (*6*)*-Id* genes detected in 2 genomes (2/5). In addition, *aadA5*, *aac* (*3*)*-IId*, *aac* (*3*)*-IV*, and *aac* (*6′*)*-Ib3* genes were found in one genome (20%, 1/5). All *E. coli* genomes harbored a *tet* (*A*) gene encoding tetracycline resistance. One isolate (BD7) harbored both *tet (A)* and *tet (M)* genes. The predominant sulfamethoxazole resistance gene was *sul3* (4/5) followed by *sul2* (1/5). Among the 4 different trimethoprim resistance genes identified, *dfrA12* was predominant (40%, 2/5). Macrolide-associated resistance gene *mph* (*A*) was commonly detected (4/5). Phenicol resistance gene *floR* was predominantly (60%,3/5) found, followed by *cmlA1* (40%, 2/5) gene. The efflux, small multidrug resistance transporter gene, *qacL*, was also detected in a poultry isolate (BD7). None of the isolates harbored carbapenemase genes and did not show phenotypic resistance to carbapenem antibiotics. Overall, the average number of AMR genes per genome was highest in poultry *E. coli* compared to human *E. coli* isolates (Table 2). The probable genome locus of the *bla*_TEM1B_ and *bla*_CTX-M_-group genes were plasmids for two strains (Table 3). In BD-Cl10 isolate, the *bla*_CTX-M_ gene was found on a chromosome with insertion element ISEcp1. The *bla*_TEM1B_ gene in the BD8 strain and BD-Cl10 isolate was linked with insertion elements ISKra4 and IS6100R, respectively (Table 5). We identified amino acid substitutions in *gyrA* at codon positions S83L (4/5) and D87N (1/5), and in *parC* at S80I (2/5), S80R (1/5) and A56T (1/5). There was a significant correlation between the *gyrA* S83L mutation and resistance to ciprofloxacin. The ESBLs genes *bla*_TEM1B_, *bla*_CTX-M_-group, and *gyrA* S83L were associated with H_2_S-producing *E. coli* strains. Additionally, all the isolates harbored PMQR genes, including *Qnrs1* (2/5), *Qnrs4* (1/5) and *Qnrs13* (1/5). The *Qnrs13* gene in the BD8 strain and *Qnrs4* in the BD-Cl10 strain consisted of the insertion element ISKra4 (Table 3). The β-lactamase genes, PMQRs and QRDRs were all strongly associated with the MDR phenotype.

## Discussion

Production of hydrogen sulphide (H_2_S) is seen in many members of *Enterobacteriaceae*. However, it is well established that *E. coli* strains are H_2_S non-producers. H_2_S non-production is one of the key characteristics used to identify *E. coli* in laboratory tests. Nonetheless, a fraction of H_2_S-producing *E. coli* variants has previously been identified in animal and human infections (Maker and Washington, 1974; Sogaard, 1975; Magalhães and Vance, 1978). By studying H_2_S-producing *E. coli*, researchers can better understand the biology and behavior of such variants and develop improved diagnostic tests. Further, a comprehensive characterization of H_2_S-producing *E. coli* including analysis of genomic features was needed. To address this, we conducted a thorough investigation of four H_2_S-producing *E. coli* variants by utilizing whole-genome sequencing (WGS) in combination with comprehensive microbiological and biochemical testing.

The four bacterial isolates were recovered from poultry and human clinical samples in Dhaka, Bangladesh, as part of a larger surveillance study. These isolates biochemically mimic typical *E. coli* for all reactions except for one reaction, the H_2_S production. The prevalence of H_2_S-producing variants in our study can be estimated at 3% (3/96) in poultry and 0.5% (1/204) in clinical *E. coli* isolates. However, this may not reflect the true prevalence figures, as in this study, the primary specimens were not screened targeting H_2_S-producing *E. coli*. But only the archived *E. coli* isolates were tested. However, our estimates of prevalence are similar to those previously reported (Maker and Washington, 1974; Sogaard, 1975; Weber et al., 1981). Our and other reports reveal that H_2_S-positive strains of *E. coli* are not uncommon among poultry and human clinical samples (Braunstein and Mladineo, 1974; Maker and Washington, 1974; Sogaard, 1975; Traub and Kleber, 1975; Magalhães and Vance, 1978; Weber et al., 1981; Barbour et al., 1985). Many such variants are probably misidentified in laboratories, such as *Citrobacter*, *Arizona* and *Salmonella* (Darland and Davis, 1974). This misidentification stems from the production of black precipitate on KIA or TSI medium. It is also possible that acid production sometimes masks H_2_S production due to lactose fermentation (Magalhães and Vance, 1978). Muller-Hinton agar supplemented with sodium thiosulfate and ferric ammonium sulfate media is considered superior to KIA agar media for identifying H_2_S production. The utility of the same has been demonstrated in this study. However, the CHROMagar Orientation media could not differentiate between typical *E. coli* and H_2_S-producing *E. coli* variants. Primary screening with this media can effectively screen typical *E. coli* and H_2_S-producing *E. coli* variants in a single step.

The studied H_2_S-producing *E. coli* strains mainly belonged to the commensal phylogenetic groups A (80%,4/5) and B1 (20%, 1/5). Several reports confirm that phylogroups A and B1 were the most prevalent among *E. coli* isolates, particularly in the gut microbiome (Li et al., 2010; Stoppe et al., 2017). The H_2_S-producing *E. coli* strains of human origin, isolated from Bangladesh and China, belonged to the worldwide predominant clonal complex CC10. CC10 group of strains belong to emerging clone of extra-intestinal pathogenic *E. coli* (ExPEC) (Manges et al., 2019). They are isolated from a wide range of niches including clinical settings, food animals and environment (Manges et al., 2019). They are also known to be associated with wide range of AMR and virulence genotypes (Massella et al., 2021). This group of *E. coli* needs close monitoring to safeguard public health (Hussain et al., 2023). We identified 8–13 prophage regions in H_2_S-producing *E. coli*, of which 2–4 were found intact. *Klebsiella* phage 4 LV-2017 and Shigella phage SfII were the predominant bacteriophages detected. The existence of a higher number of phage elements (8 to 13) in poultry strains compared to the human clinical strain (5) may indicate more horizontal gene transfer (HGT) events that brought in more toxin genes in poultry strains than in the human clinical strain. The CRISPR-Cas system confers immunity against viruses and plasmids (Horvath and Barrangou, 2010). Investigation of the CRISPR-Cas system in H_2_S-producing *E. coli* strains indicated that it was conserved in both poultry and human clinical H_2_S-producing *E. coli* isolates.

Previous work has identified cysteine-degradation genes in H_2_S-producing bacteria and classified them into primary, secondary and erroneous categories based on their functions (Braccia et al., 2021). Most primary producer genes (*aspC, dcyD, sseA, yhaOM* operon) were present in all H_2_S-producing *E. coli* strains. In the case of secondary producer genes, we observed inconsistent results. But all erroneous genes were present in the study isolates. We found all genes related to H_2_S production on chromosomes, which is in line with the previous report (Braccia et al., 2021).

The patterns of antibiotic resistance were similar for human and poultry isolates. High resistance rates were observed for ampicillin, ciprofloxacin, nalidixic acid, trimethoprim and sulfamethoxazole, doxycycline and cephalosporin. Our findings show partial agreement with the previous report on H_2_S-producing *E. coli* (Braunstein and Mladineo, 1974; Maker and Washington, 1974; Sogaard, 1975; Traub and Kleber, 1975; Magalhães and Vance, 1978; Weber et al., 1981; Barbour et al., 1985; Park et al., 2015). The H_2_S-producing *E. coli* isolates contained multiple plasmids. The major replicon types were IncX (4/5; 80%) and IncF (3/5; 60%). As per earlier reports, these plasmid replicons were associated with fluoroquinolone resistance and *bla*_CTX-M-group_ in humans and livestock *E. coli* (Phan et al., 2015; Sun et al., 2017). As healthy animals and humans were found to harbor H_2_S-producing *E. coli* (Sogaard, 1975; Biswas et al., 2020), the presence of these plasmids may contribute as careers of antibiotic resistance in microbiomes. The results of our study suggests that aminoglycosides and carbapenem antibiotics are effective candidates against these strains. However, this cannot be generalized due to several limitations of our study and it is always better to initiate evidence-based treatment of diseases arising from infectious agents.

All isolates were predicted as human pathogens as per their pathogenicity score determined by *in silico* analysis. The studied H_2_S-producing *E. coli* isolates harbored at least 30 virulence factors. Among them, poultry isolates had more virulence genes (40–57 VFs) than human samples. The H_2_S-producing *E. coli* isolates harbored a wide range of virulence factors encoding *E. coli* laminin-binding fimbriae (ELF) (*elfA,C,D,G*), Hemorrhagic *E. coli* pilus (HCP) (*hcpA-B*), Type I fimbriae (*fimD, fimF, fimG, fimH*) and Non-LEE encoded TTSS effectors (*espL1, espL4, espR1, espX4*). The intimin (*eae*) gene, a marker for enteropathogenic *E. coli,* was observed in one H_2_S-producing *E. coli* isolate (BD8) belonging to ST189. This indicates that *E. coli* pathotypes also exhibit H_2_S production features or vice versa. Therefore, virulence genes play an important role in the pathogenicity of H_2_S-producing *E. coli* strains. Also, the convergence of wide range of AMR and virulence genotypes is a cause of great concern (Massella et al., 2021). These observations warrant studying the role of H_2_S-producing *E. coli* isolates in different infections for developing effective treatments and preventive measures.

In conclusion, this study investigated H_2_S-producing *E. coli* variants recovered from poultry and human clinical samples in Dhaka, Bangladesh. The isolates were confirmed as *E. coli* by routine biochemical tests and WGS-based species identification. The H_2_S-producing isolates exhibited relatively diverse molecular characteristics with no correlation between the source of origin or the phylogenetic clustering of the isolates. The study also found high rates of AMR and extensive virulence gene repertoire in these isolates. The findings of this study highlight that the genomic features, antibiotic resistance and virulence potential of H_2_S-producing *E. coli* resemble the typical *E. coli* forms. Therefore, we suggest the need for continued surveillance and genomic characterization of atypical *E. coli* forms like H_2_S-producing *E. coli* to better understand the characteristics of such variants and improve diagnostics and treatment outcomes.

## Data availability statement

The datasets presented in this study can be found in online repositories. The names of the repository/repositories and accession number(s) can be found below: https://www.ncbi.nlm.nih.gov/genbank/, PRJNA882002, PRJNA714244.

## Author contributions

RM designed the study and conducted all microbiological tests, and whole genome sequencing. RM and AH carried out the bioinformatics analyses interpretation of results, prepared tables and figures, and drafted the manuscript. MR and RM performed the sample collections and initial sample processing. AH contributed to the discussions, manuscript writing, editing, and proofreading. AA, JP, SC, TC, and DM contributed to the discussions and reviewed the manuscript. DM managed the funds and supervised the study. All authors contributed to the article and approved the submitted version.

## Funding

The work was funded through a Royal Society International Collaboration Award (ref. ICA\R1\191309).

## Conflict of interest

The authors declare that the research was conducted in the absence of any commercial or financial relationships that could be construed as a potential conflict of interest.

## Publisher’s note

All claims expressed in this article are solely those of the authors and do not necessarily represent those of their affiliated organizations, or those of the publisher, the editors and the reviewers. Any product that may be evaluated in this article, or claim that may be made by its manufacturer, is not guaranteed or endorsed by the publisher.

## Footnotes

^1^ https://enterobase.warwick.ac.uk/

^2^ http://omics.informatics.indiana.edu/CRISPRone
